# “I Was Confident From the Bottom of My Heart That I Will be Fine With These Medicines*”*: Qualitative Analysis of Decision‐Making Around Self‐Managed Abortion Trajectories in India

**DOI:** 10.1111/sifp.70015

**Published:** 2025-05-14

**Authors:** Caila Brander, Caitlin McKenna, Caitlin Gerdts, Balasubramanian Palanisamy, Anoop Jain, Laura Jacobson, Katherine Key, Sruthi Chandrasekaran, Ruvani Jayaweera

## Abstract

While the incidence of self‐managed abortion (SMA) in India is well‐documented, why the majority of abortions in India are self‐managed remains largely unanswered. This qualitative study explores factors that contribute to decision‐making about SMA in India. Between January and August 2022, we conducted 43 in‐depth interviews with people who self‐managed abortions across six Indian states, recruiting via accredited social health activists, clinic sampling frames, and social media posts. Underpinned by the Coast et al. framework, we coded and analyzed transcripts using thematic analysis and then organized factors that contributed to people's decision‐making around SMA versus facility‐based care. Contributing factors to people's decisions not to seek facility‐based abortion care included concerns about poor treatment by providers, the unaffordability of private facility care, and fear of procedural abortion methods. Factors contributing to people's decision to seek SMA included having prior SMA experience, access to information about SMA, affordability, privacy, accessibility, and convenience. SMA is a valued option for abortion seekers in India due to perceived benefits and a desire to avoid facility‐based care. Our findings highlight the need for improved person‐centered abortion care at facilities and offer potential avenues for developing supportive resources for people who self‐manage abortion in India.

## INTRODUCTION

Self‐managed abortion (SMA), defined here as the use of abortion medications without clinical supervision (World Health Organization 2022), is common around the world, even where abortion services are legally available within healthcare systems (Sorhaindo and Sedgh 2021; Khezri et al. 2023). Across contexts, individual reasons for and pathways to SMA are influenced by a range of often intersecting factors, including barriers to facility‐based care, and personal preferences (Moseson et al. 2020; Iyengar et al. 2016; Srivastava et al. 2019). Financial concerns, educational pursuits, family planning decisions, and lack of partner support are commonly cited personal factors in the global literature (Moseson et al. 2020). Logistical challenges (travel distance, time off work, childcare arrangements), legal restrictions, cost barriers, and provider refusals present significant obstacles to obtaining facility‐based care. Additionally, privacy concerns, fear of mistreatment by facility staff, desire to avoid abortion stigma, and concerns about intimate partner violence also contribute to the reasons for self‐management of abortion among some people.

Many individuals also affirmatively choose self‐managed abortion (SMA), valuing the autonomy, privacy, flexibility, and, for some, familiarity that the self‐management process affords. The increasing accessibility of abortion pills in many global settings, positive personal or social network experiences with successful self‐managed abortions, perception of greater safety compared to facility‐based care or procedural abortion, ability to have support person presence, and the often lower costs of medication abortion (MA) in self‐managed compared to facility‐based settings all contribute to such preferences (Moseson et al. 2020). For these individuals, SMA represents not merely a fallback option when facility‐based care is unavailable, but a proactive and preferred choice.

Despite a relatively permissive legal framework, the largest number of self‐managed abortions in the world occur in India. The most recent abortion estimates (from 2015) suggest that 11.5 million MA occurred outside of health facilities (i.e., SMA), representing 73 percent of all abortions in India (Singh et al. 2018). In order to more fully understand the practice of SMA globally, exploring people's experiences with SMA in India is critical. There remains, however, little information about people who self‐manage abortions in India. To understand why so many utilize SMA when facility abortions are legally available, a closer look at the legal framework and application of legal and clinical guidelines is necessary. In India, The Medical Termination of Pregnancy (MTP) Act was passed in 1971 and allowed for the provision of abortion services up to 20 weeks gestation under certain circumstances, including to preserve the pregnant persons’ mental and physical health, due to economic or social necessity, and in the case of contraceptive failure for married couples (Ministry of Health and Family Welfare, Government of India 1971). In 2003, the abortifacient medication, mifepristone, was approved for abortions through 12 weeks gestation (Ministry of Health and Family Welfare, Government of India 2003). More recently, MTP provisions were clarified to include instances of contraceptive failure for unmarried women and abortions between 20 and 24 weeks of gestation under some circumstances (Banerjee and Andersen 2012; Arora and Singh 2022; Kalyanwala et al. 2010). Throughout the evolution of the MTP Act, the government has maintained restrictions on who is legally allowed to prescribe and provide MA care, limiting the authority to provide abortion care to “registered medical practitioners” as stipulated by the MTP Act (Ministry of Health and Family Welfare, Government of India 2003). Practically speaking, this means that in India, abortions can be performed in registered facilities by obstetrician‐gynecologists or by doctors with bachelor's in medicine or surgery who are specifically trained and certified to provide abortion care (Stillman et al. 2014). The demand for abortion in India exceeds the capacity of the number of trained, certified, and willing abortion providers and facilities providing abortion care (Bhate‐Deosthali and Rege 2019; Singh et al. 2018). There is particularly limited availability of abortion services in health facilities within rural areas (Moore et al. 2019; Pyne and Ravindran 2020).

The MTP Act requirements may compound cultural and practical barriers that can put abortion care out of reach, particularly for marginalized communities. Health facilities with practitioners legally authorized to provide abortion care may not be accessible or desirable for a variety of reasons (Iyengar et al. 2016), and abortion seekers may face discrimination from providers who may delay or refuse care on the basis of age, marital status, economic circumstances, and other characteristics (Acharya and Kalyanwala 2012; Center for Reproductive Rights 2018; Chandra et al. 2021; Sunil 2022; L. Jacobson et al. 2023). Although the government has aimed to facilitate access to abortion by making it available free of charge through the national network of government‐run public health facilities, many provide substandard care or do not provide abortion care at all (Stillman et al. 2014; Pyne and Ravindran 2020; Shekhar et al. 2020; Singh et al. 2020). Most facility‐based abortion provision happens through the private health sector (Singh et al. 2018; International Institute for Population Sciences and ICF 2022), which can be cost‐prohibitive and less accessible in rural areas (Shekhar et al. 2020; Sharma and Pradhan 2020). Across the public and private sectors, an estimated one in three procedural abortions is performed using dilation and curettage (Singh et al. 2020), which the World Health Organization no longer recommends compared to medical vacuum aspiration (World Health Organization 2021). Those who desire MA from public or private facilities may find that abortion medication is unavailable (Singh et al. 2020) or that it requires multiple visits (Iyengar et al. 2016), creating barriers to access.

Despite the legal stipulation that people must have a prescription from a registered medical provider to receive abortion medications, pharmacy provision of abortion medication to those who do not have a valid prescription is common in India (Diamond‐Smith et al. 2019; Powell‐Jackson et al. 2015). Research has shown that chemists and pharmacists are the primary source of access to MA and abortion information in India, and that, most commonly, it is the male partner of the person seeking the abortion who procures the abortion medications at the pharmacy (Diamond‐Smith et al. 2019; Srivastava et al. 2019). Studies on pharmacy provision of MA, most of which is over‐the‐counter, have documented significant gaps in the quality of information on regimen dosing, what to expect, and side effects, as well as discriminatory prescribing practices on the basis of personal characteristics such as marital status along with challenges in delivering information and protocols to a client (i.e., the male partner) who is not the abortion‐seeker themselves (Srivastava et al. 2019; Sunil 2022; Diamond‐Smith et al. 2019; Percher et al. 2021).

Stigmatizing attitudes towards abortion and SMA have also been documented in the literature around abortion access and experiences in India. In one study of people who SMA, participants reported feeling constrained by negative perceptions around abortion by partners/husbands, mothers‐in‐law, physicians, and pharmacists. These perceptions resulted in some participants keeping their abortions secret in order to avoid judgment (Purple Audacity Research and Innovation Private Limited 2020). Abortion stigma has also been documented as having influence on the decision about which pharmacy to approach in order to procure MA, for example, choosing a shop where no other clients were present, or visiting a pharmacy that is some distance from their homes to avoid recognition (Srivastava et al. 2019).

Stigma surrounding abortion in India, coupled with the fact that those obtaining medications from pharmacies are often male partners, not abortion seekers themselves, means that documenting the experiences of individuals who self‐manage their abortions in India presents both practical and methodological challenges. As a result, much of the published research on SMA in India has used facility‐based recruitment, which highlights the perspectives of those seeking postabortion care but may not represent the full scope of SMA experiences (Srivastava et al. 2019; Banerjee and Andersen 2012; Arora and Singh 2022; Kalyanwala et al. 2010; Pawde, Ambadkar, and Chauhan 2016; Ganatra et al. 2010). Studies that have employed community‐based recruitment strategies in India have often illuminated experiences across multiple abortion modalities rather than exclusively SMA (Srivastava et al. 2019; Sunil 2022; Bowling et al. 2021). Researchers have indicated the need for more qualitative exploration into the reasons and context surrounding the decision to self‐manage abortion over other methods and modes of service delivery (Goemans et al. 2023). A better understanding of pathways to and decision‐making around SMA could have implications for improving the quality of abortion care offered in facilities and highlight opportunities to support those self‐managing their abortions.

In this study, we aimed to contribute nuanced, qualitative research on people's SMA experiences to a growing evidence base about SMA in India. In our analysis, we assessed factors that influenced the decision‐making around the model of abortion‐related care (SMA vs. facility‐based abortion) through the lens of the Abortion Trajectories Framework (Coast et al. 2018). Coast et al.’s framework presents a range of factors that influence trajectories towards abortion‐related care in three domains: (1) *Abortion‐specific experiences*, which capture time‐dependent processes involved in abortion care from pregnancy detection through steps taken to obtain abortion care; (2) *individual context*, which capture personal characteristics that may influence one's abortion seeking and experience such as knowledge and beliefs, interpersonal relationships, and agency; and (3) *(inter)national/subnational contexts*, which capture the broader structural, legal, and health systems contexts as well as the broader knowledge environment and sociocultural factors. Abortion trajectories are complex; a variety of factors from all three framework domains may exert influence at different points along one individual's abortion experience. In this paper, we explore a range of pathways to and decision‐making around SMA for abortion seekers in India, with a specific focus on factors that deterred people from seeking facility‐based care and/or motivated them to pursue SMA.

## MATERIALS AND METHODS

### Data Collection

From January to August 2022, we recruited people living in six states in India who had self‐managed medication abortions in the last five years to participate in a qualitative study about their experiences. Participants were eligible if they were 18 years or older; spoke English, Hindi, Tamil, or its dialects; self‐managed an abortion using abortion medications (misoprostol alone or in combination with mifepristone) obtained without a prescription within the last five years (2017–2021) and provided verbal consent to participate in the study.

The study team conducted a rapid review of the literature, using the abortion trajectories outlined in the Coast et al. framework (Coast et al. 2018) to identify research gaps in the current literature on each stage of the abortion trajectory in India (Chandrasekaran et al. 2021). Research gaps included how people chose SMA, the information they had about SMA before having their abortion, what their abortion experience was like, and more. We then designed an in‐depth interview guide (see Online Appendix I) to document and explore diverse pathways, decision‐making, and experiences with SMA in India. The gaps identified in the SMA literature in India and the Abortion Trajectories Framework informed the development of the in‐depth interview guide, which was designed to touch upon each stage of the abortion trajectory including individual contextual factors, national and subnational factors such as sociocultural context, and recommendations from people who self‐managed abortions for improving abortion care. The interview guide was developed in English. A professional firm translated the guide into Hindi, and a recruitment site translated it into Tamil. During training on interview techniques and abortion values clarification, members of the study team and interviewers reviewed and practiced the interview guide in their respective language to ensure consistency with the original text and appropriate terminology in each language. The study team and interviewers iterated the guide for question clarity, and additional probing was added following the first and second interviews at each site, then the guide was finalized for the remainder of data collection.

Our recruitment used four distinct strategies, aiming to capture diverse experiences based on geography, participant characteristics, and abortion experiences, including follow‐up care‐seeking. First, India‐based sexual and reproductive health organizations posted study information on social media. Second, Accredited Social Health Activists (ASHAs) in Jharkhand and Bihar informed their communities about the study and referred potentially eligible individuals interested in participating in the study to interviewers for screening. Third, a community‐based organization in rural Tamil Nadu recruited from a sampling frame of people who had received nonabortion care through their clinic in rural Tamil Nadu. Fourth, we recruited from a sampling frame of individuals who sought postabortion care at three clinics in Madhya Pradesh, West Bengal, and Maharashtra who had participated in a separate study and agreed to be contacted about other study opportunities. During recruitment, 127 people were approached or screened by the study team; 81 refused or were ineligible and did not complete an interview. Three completed interviews did not meet eligibility criteria upon review and were excluded. A total of 43 people participated in the in‐depth interviews. The final sample size was determined by multiple factors: hypothesized thematic saturation on key areas of the abortion trajectory, inclusion of diverse perspectives from each recruitment site, and the budget. Where possible, we purposively sampled to ensure diversity in terms of age, caste, religion, and marital status. This included intentional recruitment among historically marginalized communities in Jharkhand, Bihar, and Tamil Nadu. More in‐depth recruitment information can be found in Online Appendix II.

Participants provided verbal consent and were interviewed face‐to‐face, where feasible based on interviewers’ locations (Jharkhand, Bihar, and Tamil Nadu), and over Zoom at other recruitment sites. In‐person interviews were conducted by —two to four trained Indian female interviewers per site, while one Indian female interviewer completed all virtual interviews. Each participant was compensated with a Rs. 700 (∼8.50 USD) cash voucher or gift card. Recordings in Tamil or Hindi were translated and transcribed directly into English by a professional firm. Recordings in English were transcribed verbatim by the same firm. Transcripts were reviewed by recruitment sites for quality assurance and to remove any identifying information. Two study team members (CB and SLC) reviewed all de‐identified transcripts for clarity and resolved ambiguities with the transcription firm.

### Analysis

Two study team members (CB and SLC) developed a preliminary codebook using a blend of deductive themes generated from the Abortion Trajectories Framework to structure the in‐depth interview guide (Coast et al. 2018) and inductive themes that emerged from reviewing a subset of three transcripts from different recruitment sites (Bryant and Charmaz 2007). Four study team members (CB, CM, SC, SLC) independently coded the same transcripts and met to discuss agreement and meaning as well as to determine relevant codebook revisions based on discrepancies. These iterative sessions resulted in redefining codes, adding new codes, and collapsing codes where necessary until a final codebook was agreed on. Four study team members (CB, CM, SLC, KK) applied the final codebook to all transcripts in MAX QDA (VERBI Software, MAXQDA 2022, software, 2021, maxqda.com).

We used qualitative thematic analysis to analyze the data (Vaismoradi et al. 2016). Two study team members (CB and CM) summarized excerpts in analytic memos and met biweekly to identify relationships between code summaries and determine additional codes to summarize. We mapped findings from analytic memos into themes related to how participants made SMA decisions. The study team provided input on analytic memos as they were developed and helped iterate decision‐making themes. In this analysis, we organize decision‐making themes in alignment with components of the Coast et al. framework that contributed to reasons abortion seekers proactively pursuing SMA and factors that deterred abortion‐seekers away from facility‐based care. Each decision‐making theme is presented with an indication of the corresponding domain (i.e., abortion‐specific experiences, individual context, or (inter)national/subnational context). Similar framing has been used by others describing healthcare decision‐making (Nyakang'o and Booth 2018) including abortion (Arambepola and Rajapaksa 2014).

In this paper, we share findings that emerged on participants’ reasons for abortion, information sources, prior abortion experiences, and barriers and facilitators to abortion care. We attribute quotes by age, state of residence, rural versus urban residence, and marital status. A robust reflexivity statement for the study team can be found in Online Appendix III. The study protocol and all instruments were reviewed and approved by Sigma Institutional Review Board (New Delhi, India).

## RESULTS

### Sample Characteristics

We conducted in‐depth interviews with 43 people aged 19–47. Most participants were married (*n* = 40) and lived in rural areas (*n* = 30). More than half of the participants were recruited from Jharkhand and Bihar. About a third of participants were from a religious minority group (*n* = 15) and had more than one abortion experience (*n* = 16). The median price for the abortion medication was Rs 500 (∼6.00 USD) and ranged from Rs 50 to Rs 4000 (∼0.60 to 48.00 USD).

Based on verbal descriptions of the medications procured, almost all participants (*n* = 35) received the combi‐pack of one mifepristone and four misoprostol, whereas five participants described receiving a different number of pills, and in three cases it was not clear what number of pills or which medication(s) were received. The medication regimens used by participants varied, with only one participant precisely following the WHO‐recommended regimen. Most participants described regimens that involved varying numbers of pills, time between doses, and non‐WHO‐recommended administration routes for misoprostol (primarily oral). Prior to using the medications, seven participants first attempted to end their pregnancy using a traditional method, such as eating papaya, pineapple, cumin seeds, coffee, or taking deworming medication. All participants in our study had a complete abortion before 12 weeks of gestation. These data are summarized in Table 1.

**TABLE 1:** Characteristics of a sample of self‐managed abortion users in India who participated in in‐depth interviews for this study (*N* = 43)

Participant characteristics	*n* (%)
Age	
18–24	14 (33)
25–34	22 (51)
35+	7 (16)
Marital status	
Married	40 (93)
Unmarried (i.e., never married)	3 (7)
State ^a^	
Tamil Nadu (rural)	4 (9)
Jharkhand (rural)	13 (30)
Bihar (rural)	9 (21)
West Bengal (rural)	3 (7)
Maharashtra (urban)	3 (7)
Madhya Pradesh (peri‐urban)	8 (19)
Unknown state (two urban and one rural)	3 (7)
Number of children	
None	3 (7)
One	10 (23)
Two	17 (40)
Three	7 (16)
Four	2 (5)
Five or more	4 (9)
Number of abortions prior to most recent SMA	
None	27 (63)
One	10 (23)
Three	4 (9)
Four	2 (5)
Year abortion of interest for the study took place	
2022	13 (30)
2021	17 (40)
2020	9 (21)
2019	3 (7)
2018	1 (2)
Religion	
Hindu	14 (33)
Muslim	14 (33)
Sikh	1 (2)
Missing	14 (33)
Education level	
Not formally educated	6 (14)
At least some primary	8 (19)
At least some secondary	22 (51)
At least some undergraduate, postgraduate diploma, or graduate school	7 (16)
Caste (condensed)	
General Caste	4 (9)
Scheduled Caste/Scheduled Tribe	9 (21)
Other Backward Caste	30 (70)

^a^Geographic location was based on recruitment site: participants from Tamil Nadu were recruited through a non‐abortion clinic client sampling frame; those from Jharkhand and Bihar were recruited by ASHAs; users from West Bengal, Maharashtra, and Madhya Pradesh were recruited at clinics from those seeking postabortion care; users with unknown locations were recruited online, where we asked if they lived in rural, urban, or peri‐urban areas but did not collect specific geographic information to preserve their anonymity.

In pursuing self‐managed abortions using medications, participants weighed a variety of factors that either proactively motivated abortion‐seekers to pursue SMA or deterred them from facility‐based care. Abortion method options were often discussed, and decision‐making was shared between those having an abortion and their family members, such as their husbands, mothers, and mothers‐in‐law. The contributing factors described in this analysis center on the perspectives of participants (the individual having the abortion) on why they pursued SMA over facility‐based care.

The findings are presented in the following order: (1) factors contributing to decision‐making about abortion‐related care (SMA vs. facility‐based care), (2) motivating factors towards SMA, and (3) reflection on SMA decision‐making and experiences. Each theme is aligned with its corresponding domain from the Abortion Trajectories Framework (abortion‐specific experiences, individual context, or (inter)national and subnational context).

### Factors That Deter Abortion Seekers From Pursuing Facility‐Based Abortion Care?

Factors that deterred abortion seekers from pursuing facility‐based care included concerns about poor quality treatment by facility staff, loss of confidentiality, and the cost of private facility‐based care as well as fear, lack of information, and misinformation about procedural methods.

#### (Inter)National and Subnational Context: Concerns About Poor Treatment and Confidentiality Loss During Facility‐Based Care

Participants were concerned about low‐quality care and loss of confidentiality at facilities, sometimes based on negative past experiences. This was particularly true for public health facilities, which were generally perceived as less desirable than private health facilities.

Participants believed that public health facility staff and providers lacked sensitivity and confidentiality. One shared, “[in the] public health facility they talk a lot if we go, they judge us also” (29 years old, Tamil Nadu, rural, married). Participants shared that perceptions of poor‐quality treatment were pervasive in their communities. Some participants had already experienced poor treatment when seeking healthcare at public health facilities, including abortion care. Many referenced scolding, whereby hospital nurses or doctors shamed patients. As this mistreatment was reportedly common for all types of medical services at public health facilities, participants in communities with increased abortion stigma understood that the risk of scolding, judgment, and confidentiality breaches was more severe for abortion patients:

I know that public health facilities are cheap, but they are public health facilities. So, if anything happens, they will tell the cops, that goes without saying. And there's a lot of moral policing from what I've heard. (20 years old, urban, unmarried)

A few participants shared that they feared judgment in private facilities as well, especially if they felt they had “transgressed,” from the provider's point of view, by not preventing their pregnancy: “If [at the private facility] I meet the same doctor, then he would think why am I coming there again and again as he told me to use birth control methods” (28 years old, Tamil Nadu, rural, married).

Some participants expressed wanting to avoid facilities where they could not be seen immediately or privately by a provider, which was typically associated with seeking care at a public health facility. The anticipation of long wait times could deter seeking care when participants needed to be accompanied to a hospital by their husband or male relative or felt overwhelmed with household duties like childcare:

I have three children. I can't take them to the doctor [with me]. We have to wait at the public health facility, and it takes a lot of time. It takes an entire day. So I can't take my children there and wait. We go [to the private facility] if it is serious…. (26 years old, Jharkhand, rural, married)

Some participants also worried about the attention that an extended absence from their normal tasks, while waiting for care, would draw from others in their community: “People [would] ask me why I am going there. That is why I didn't go to hospital” (26 years old, Tamil Nadu, rural, married).

#### Individual Context: Private Facility‐Based Care Is Desirable but Costly

Care from private facilities, while described as more desirable than from public health facilities, was cost‐prohibitively expensive for many. One participant explained that “if we had money, then we could have gone to the [private] doctor—but we don't have the money, so I had the [abortion] medicine” (22 years old, Jharkhand, rural, married). Another explained, “I didn't go to the [private] doctor because we all have financial issues here. If we go to the [private] doctor, then more money will be needed” (23 years old, Bihar, rural, married). Some shared that to afford care at a private facility, they would be forced to neglect other financial responsibilities and therefore negatively affect their families:

If I go to the private doctor, then they … charge Rs. 3,000. How can I pay that money? The income that I have is Rs. 3,000 per week. If I give that to the doctor, then what can I do to run the family? So, I thought of aborting using the tablets. (26 years old, Tamil Nadu, rural, married)

#### National Context and Individual Abortion Experiences: Fear of Procedural Abortion

In the Indian context, as well as globally, procedural abortion methods, which could include WHO‐endorsed procedures such as manual vacuum aspiration or regimens no longer recommended for abortion, such as dilation and curettage, are primarily administered in facilities (Bell, Shankar, and Moreau 2021). While not all participants were aware of procedural methods, those who were often expressed fearing this method. Some described fearing aspects of medical interventions, such as injections or machines: “I don't feel good in the public health facility… I am scared of injections” (26 years old, Jharkhand, rural, married). Others worried about the pain of a procedural abortion:

If we go to a public health facility, then I cannot tolerate that pain of D&C [dilation and curettage]. They do some procedures, and I cannot tolerate that pain. Already my body is damaged. If I go there, then they do scary procedures. (26 years old, Tamil Nadu, rural, married)

The belief that participants would experience severe pain if they chose this method was partially derived from portrayals of abortion procedures as unsafe:

[M]y only apprehension was that I didn't want to go through the surgery method. Also, because through movies and social media, I had always perceived a surgical abortion as a very, very dangerous thing. I did not consider it to be a safe option to begin with. (28 years old, urban, unmarried)

### Motivating Factors Towards SMA

Several aspects of self‐managing an abortion, including prior experience with SMA, greater availability of information about SMA, affordability, the opportunity for greater privacy, convenience, and, in some cases, limited ability to access other choices, made SMA an appealing option for abortion‐related care.

#### Abortion‐Specific Experiences: Prior Experience with MA and/or SMA

Several participants had prior MA, both with and without clinical supervision, and drew upon these experiences to guide their subsequent abortion decision‐making. Participants with prior MA already knew about abortion medication from health workers or family members and had confidence about the safety and effectiveness of the medication. After going to the doctor for her first abortion and being recommended abortion medication, one participant said, “from then on, whenever needed, [I] kept taking the medicine.” (36 years old, Jharkhand, rural, married).

For those who had self‐managed a prior abortion, knowing where to obtain medications contributed to their decision to self‐manage again. For her first abortion, one participant had received a referral from a local provider (LP) to a pharmacy; when she needed a subsequent abortion, her husband went directly to the same pharmacy.

#### Individual Context: SMA Is Well‐Known Compared to Other Methods

The information available to participants made SMA more appealing. Some participants lacked information about the full range of abortion options, only learning about procedural methods after their SMA: “I was not aware of this [procedural methods] at that time. Now I know about it, but at that time, I did not know” (28 years old, Tamil Nadu, rural, married).

Of those who were aware of procedural methods, none considered going to a facility to receive abortion medications. Rather, participants characterized their choice as a decision between a procedural abortion in a facility or SMA procured outside of health facilities. As one participant said, “Just two [abortion methods] are there, like either take the [abortion] medicines or go to the doctor” (30 years old, Jharkhand, rural, married). Another participant reiterated this idea that self‐managing using medication or a procedural abortion from a facility were their only options to choose from

[T]he [abortion] pill was the only option. See, because it's not like I had given two paths, and I could choose – one led to a doctor and one led to a pill; there was just one path for me to take. (24 years old, rural, unmarried)

It was unclear if participants did not mention the possibility of getting an MA at a facility due to preferences, other barriers like financial concerns, or lack of awareness. Overall, the lack of information about alternative modalities of care or methods available meant some women only considered SMA, as one participant concisely expressed, “I didn't think of any other way” (29 years old, peri‐urban, married).

In contrast, information about SMA from peer networks drew people towards SMA. Many participants reported that abortion medication was well‐known in their communities. Some participants knew close friends or family who had previously used medications and could provide additional insight about the experience. This facilitated SMA use: “Everybody takes [abortion] medicines, so I thought I will also try it out” (22 years old, Jharkhand, rural, married). Another stated,

Everyone told me that things get fine with this. I wanted to check it out as I have never done this before and if I succeed, then it will be good. Why go to a doctor? That's why… I didn't think so much. I was confident from the bottom of my heart that I will be fine with these medicines. That's why I took them. (28 years old, Jharkhand, married)

Before deciding on an abortion method, participants often spoke to trusted loved ones or community health workers as part of their decision‐making process. Those who had access to ASHAs or LPs described encounters as nonjudgmental mechanisms to get information and support when choosing an abortion option that was offered for free (ASHAs) or for a nominal fee from LPs. Participants described how this counseling aligned with their specific circumstances, acknowledging the economic constraints many participants faced and advising accordingly. For example, one applicant shared, “Bigger doctors are very expensive. [The ASHA] told me that [the medication abortion] will be done in this much money and [the abortion was complete]” (30 years old, Jharkhand, rural, married).

#### Individual Context: SMA Is More Affordable Than Facility‐Based Care

While most participants were not initially aware of the exact pricing of abortion medications, they understood that paying for “medicine” would be much cheaper than clinical consultation and treatment: “It is cheaper to take tablets from medical shops” (28 years old, Tamil Nadu, rural, married). Some participants who inquired about the difference in price between private facility care and MA learned that abortion medications from a pharmacy were a small fraction of the cost.

Some participants seemed to view SMA as the first, more affordable, and accessible step towards ending a pregnancy, and that they could and would escalate to facility‐based abortion care if SMA did not work:

Washing [procedural abortion] takes Rs. 5000–6000… So, the best thing is to get it [the abortion] done at home [using abortion medication]. That will be better. Usually, people visit the doctor only when something goes wrong. (40 years old, Bihar, rural, married)

When participants sought support from LPs, these consultations were either free or involved a minor fee compared to what they would pay at a private facility.

#### Abortion‐Specific Experiences: SMA Is More Discrete and Offers Greater Privacy

With SMA, participants believed they could go through the abortion process in private, without others needing to know. All participants wanted to hide their pregnancy or abortion from at least one person, such as family, partners, or their community. One of the major underlying reasons for this was perceived negative family and community attitudes about abortion. Visiting a local pharmacy was a much more discrete way to get the medications than a trip to a facility, which would have been noticed by others. One participant who did not want to share about her abortion with others said, “I clearly knew I could not go see the doctor… I knew that I had to do it [the abortion] on my own. The part of getting a pill was clear to me” (24 years old, rural, unmarried). A married participant who was not supported by her family to get an abortion shared, “I thought if I take medication, nobody will know about it. I can do things silently” (28 years old, Tamil Nadu, rural, married). Some participants who had consultations with LPs or ASHAs were able to receive abortion counseling and sometimes receive abortion medication from them within the privacy of their own homes, “He [the LP] used to come at home for the treatment like cough and cold, so I asked him [for medication abortion pills] at that time” (47 years old, Bihar, rural, married).

#### Abortion‐Specific Experiences: SMA Is More Convenient and Accessible

Many participants believed that the SMA process was straightforward and simple, including one who chose this method because it would be “easy and something I can do at home” (20 years old, urban, unmarried). Others felt this was the easiest method due to information received or perceptions that the abortion experience itself would be quick and without “hassle.” For instance, some believed that the pregnancy would be “cleared faster with medicine,” or only considered abortion medications because they did not have time to recover at the hospital from a procedural abortion.

Participants with prior abortion experience shared these opinions about the ease or convenience of SMA. One participant and her husband kept abortion medications on hand: “We keep the medicine in case anybody needs [it], then we can use the same” (36 years old, Jharkhand, rural, married). Generally, participants opted for this method in part due to the accessibility of MA at local pharmacies or with community health workers. This provided a lifeline of care for those who faced access barriers, including facilities that were far away or shut down:

Initially I thought that I should go and consult the doctor first means go to the hospital where I took my co‐sister… But that was far, and so I thought pregnancy is not that advanced and so through medications only it can be done. (30 years old, Madhya Pradesh, peri‐urban, married)

#### Individual Context: SMA Was the Only Option Available to Some

For a few participants, SMA was the only method they felt they had available to them. One participant sought SMA because she was in a violent and abusive relationship and hoped to conceal the abortion from her partner, who was less likely to notice the financial impact or time away from home for an SMA compared to the cost or time facility‐based care‐seeking would require. Unmarried abortion‐seekers in India described insurmountable barriers to accessing abortion care through the formal health system. Several described being denied care by providers. For example, one participant first sought care through a telemedicine platform. While the provider was able to offer information and counseling, they said they could not prescribe medications to the participant because they were unmarried.

### Reflection on SMA Decision‐Making and Experiences

Participants had mixed reflections on their SMA decision‐making and experiences. Some were happy with their decision to use SMA and would do it again themselves or recommend it to a friend who needed an abortion: “I will advise [others] to take medicine [SMA]. If we go to the doctor, then they scold” (23 years old, Jharkhand, rural, married). Those who wished they had made a different choice or would not recommend SMA usually connected this to stigma about SMA or lack of information about abortion method options. SMA stigma was evident when participants described interactions with healthcare workers, including pharmacists and clinical providers.

For example, one participant described feeling judged by the pharmacist and shop workers when procuring abortion medications:

I felt awful. He (the shop owner) saw me in an odd way… I can't directly go and ask in front of the shop. I have to stand at the side… So they made me stand there [for 10–15 minutes]. The others looked at me in an odd way. I felt awful with that itself… I felt very bad after going to those medical shops. (28 years old, Tamil Nadu, rural, married)

Stigma against SMA was also evident in descriptions of community attitudes and sometimes even participants’ own beliefs. Those who had internalized SMA stigma said they should not have self‐managed and instead should have done their abortion the “right way” (i.e., with provider supervision).

Some participants who were unaware of procedural abortion at the time of their abortion expressed that they would have wanted that method in retrospect. This was sometimes due to negative SMA experiences where participants had unexpected or severe side effects and feared they had a complication from the SMA. Others acknowledged that SMA does not always involve the same ongoing observation and care that patients receive in clinical settings, and felt this oversight was more desirable to have.

All participants, even those happy with their method decision, wanted a more supportive environment for those making abortion decisions in India. For example, one participant advocated for abortion seekers to have access to the full range of abortion methods to exercise their true choice:

[T]he next generation of girls should not struggle like us. They should get all the facilities. The public health facility and neighboring people should support them. (28 years old, Tamil Nadu, rural, married)

Another participant suggested greater access to community‐based medication provision:

Nurses are not giving tablets. We have to go to the public health facility or private hospital for that. We need free [medication abortion]. (29 years old, Tamil Nadu, rural, married)

Other recommendations included spreading awareness about abortion methods for more informed method decision‐making, as well as programming to address provider bias so that facility‐based options become more accessible for those who prefer it.

## DISCUSSION

Our study explored the abortion pathways, decision‐making, and experiences of people having self‐managed abortions in different regions of India. For this study, we used the lens of the Abortion Trajectories Framework to assess factors that deterred abortion seekers from facility‐based care or motivated them to proactively seek SMA (Coast et al. 2018). Some factors that deterred abortion seekers from facility‐based care included fear of mistreatment by facility staff, long wait times, concerns about privacy, concerns about stigma from providers and communities, lack of knowledge about available care, and fear of/lack of awareness of/misinformation about procedural abortion methods. While care at private facilities was generally described as more acceptable than in public health facilities, it was also cost‐prohibitively expensive for most participants in this study. Similar negative experiences with public health sector abortion services and financial constraints have been reported throughout the global literature on SMA (Moseson et al. 2020; L. E. Jacobson and Gerdts 2023). In our study, many who chose SMA did so because they feared low‐quality facility‐based care. Studies have found that providers in India hold negative attitudes towards abortion or abortion seekers (L. Jacobson et al. 2023; Hogmark et al. 2013; Banerjee et al. 2014; Pyne and Ravindran 2020) and are reluctant to provide MA or any abortion care to clients who are poor, rural, uneducated, young, or unmarried (Acharya and Kalyanwala 2012; L. Jacobson et al. 2023; Bhate‐Deosthali and Rege 2019). Findings from this study corroborate these findings, demonstrating abortion seekers in India often feel they are unable to access respectful abortion or postabortion care in health facilities (Stillman et al. 2014; Sunil 2022; Percher et al. 2021). Fears of feeling stigmatized during interactions with providers have been documented as a reason abortion‐seekers choose to self‐manage in a range of healthcare and legal contexts globally (Harries et al. 2021; Ortiz, Blades, and Prada 2024; Larrea et al. 2021).

Within India, addressing providers’ negative attitudes towards SMA and abortion generally as well as the imperative to provide judgment‐free care could make facility care a better option for abortion seekers and enable those who choose to self‐manage the option to seek acceptable postabortion care if they want or need it. People deserve access to all models of abortion care, including the option to self‐manage in addition to comprehensive, high‐quality facility‐based care, yet SMA remains highly stigmatized in the medical community (L. Jacobson et al. 2023). Destigmatization of SMA within the formal health system is an essential component of ensuring greater access to abortion care (Yanow, Pizzarossa, and Jelinska 2021).

Fear and mis/disinformation about procedural methods have also been reported in the literature, most often in highly medicalized, Western country contexts with respect to abortion method decision‐making and the influence of misinformation proliferated via online and social media platforms (Georgsson et al. 2019; Jacques et al. 2021; Kanstrup, Mäkelä, and Hauskov Graungaard 2018; Nguyen, Cartwright, and Upadhyay 2023). No other study has documented this exact phenomenon in India, but there has been extensive documentation about misinformation related to COVID‐19 and other health topics (Al‐Zaman 2021; 2022; Malki, Patel, and Singh 2024). Further exploration of the role of social media, misinformation, and opportunities for course correction with respect to people's concerns about procedural abortion in the Indian context is warranted.

In this study, participants stated a preference for MA over procedural abortion methods for a range of reasons, often because they were more familiar with MA given that they had been exposed to information and knowledge about medications for abortion, and because abortion medications are widely used and readily accessible. SMA was also described as being more private, discrete, and affordable. Preferences for self‐managing described by participants in this study, including accessibility, low cost, and opportunity for discretion and privacy, are aligned with prior research in India (Srivastava et al. 2019; Sunil 2022) and across a range of settings globally (Moseson et al. 2020) that document the ease, convenience, and discrete nature of having an abortion at home. The desire for discretion and privacy is not necessarily linked to perceived or experienced abortion stigma, but it may be, and the role of familial and social networks in decision‐making about abortion care as well as abortion experiences is critical to understanding and supporting people having abortions regardless of the method or model of care. Past research in India shows that married women and pregnant people often make reproductive decisions, particularly those involving contraception, abortion, and delivery planning, in tandem with their husbands (D'Souza et al. 2013; Kumar and Maruthakutti 2015; Maharana 2017). While it is not uncommon for husbands (or male partners) to be considered the sole decision‐makers, some prior studies have documented more participatory decision‐making with respect to abortion (Jejeebhoy et al. 2010; Ganatra et al. 2010). Prior literature also reveals that the role of mothers and mothers‐in‐law in decision‐making can vary widely; with some studies documenting mothers‐in‐law playing a strong role in whether or not their daughters‐in‐law seek abortions (Visaria et al. 2004; Kalyanwala et al. 2010) while other studies find no evidence of mothers or mothers‐in‐law playing a role in abortion decision‐making (Jejeebhoy et al. 2010; Ganatra et al. 2010). Future studies exploring the role, views, and influence of partners and family members throughout the SMA process are needed to understand the roles through the SMA journey and the specific needs to support people's self‐managing abortion.

A unique feature of this study is the legal context of SMA or abortion more broadly. In India, facility‐based abortion care is legal under a broad range of circumstances, though the MTP Act mandates that a registered medical practitioner must administer abortion medications for the abortion to be legal (Ministry of Health and Family Welfare, Government of India 1971). However, the belief that any abortion care in India is illegal is widespread among communities and providers (Srivastava et al. 2019; Stillman et al. 2014). Interestingly, illegality, either of SMA or abortion generally, was not explicitly mentioned by participants as part of their method of decision‐making. While beliefs about or realities around abortion legality may have influenced other factors participants cited (e.g., by inflaming the degree of stigma they feared from providers and others in the community members), participants seemed to make their method choice without, at least explicitly, fear of criminalization for choosing SMA.

All abortion decisions should be supported by access to comprehensive information about the full range of abortion methods and models of care (World Health Organization 2022). Our findings that participants often made abortion care decisions with limited information align with other studies in India, and in other contexts where SMA is common (Shankar et al. 2025), documenting limited access to information during abortion decision‐making (Bowling et al. 2021). In other contexts, those who self‐manage may be supported by online telemedicine services (Gomperts et al. 2008), community‐based distribution (Foster, Arnott, and Hobstetter 2017; Foster et al. 2022), and feminist accompaniment and safe abortion hotlines (Jelinska and Yanow 2018; Erdman, Jelinska, and Yanow 2018). These models of care, information, and support exist in a range of legal contexts and could be modified and/or adapted to the Indian context as well.

Our findings additionally raise the question of how to meaningfully improve trajectories to abortion care and abortion experiences for people in India. One well‐tested intervention could be to expand the provider base trained to dispense MA and/or provide pre‐ and postabortion counseling (The Lancet 2015). While the MTP currently stipulates that only registered medical practitioners (those with bachelor's degrees in medicine or surgery) can provide abortion medications (Ministry of Health and Family Welfare, Government of India 2003), pilot programs have shown community health or ayurvedically trained providers can be trained to provide abortion care to great success (Foster et al. 2022; Jejeebhoy et al. 2012; Tebbets et al. 2018). In Australia and Canada, enabling pharmacists to prescribe mifepristone has led to improved access to abortion services, especially in rural areas (Grossman and Goldstone 2015; Government of Canada 2021). Even without amending dispensing privileges, more can be done to train lower level providers to provide comprehensive counseling to address information gaps and dispel mis/disinformation in alignment with the World Health Organization's Abortion Care guidelines (World Health Organization 2022). This could help to ensure that individuals can access comprehensive abortion counseling from the providers they feel most comfortable with.

Another route to providing access to information through trusted sources is via the continued efforts of grassroots organizations. In rural Tamil Nadu, the Rural Women's Social Education Centre trains community health workers with supplemental education on sexual and reproductive health topics, including abortion, that they otherwise would not have received from their standard training (Gopichandran et al. 2024). Organizations like Hidden Pockets and Agents of Ishq have utilized art, song, and storytelling to share information about abortion in a light‐hearted and accessible way directly with young people (Agents of Ishq 2023; Hidden Pockets Collective 2023). These projects not only provide information but also address people's social contexts, emotional support, and abortion stigma, thus providing more holistic support for people's abortion experiences.

The Abortion Trajectories Framework that guided the analysis for this study was first pioneered by Coast et al. and has been utilized across a range of contexts (Larrea et al. 2021; Pleasants et al. 2024; Coast and Murray 2016) including in India (Nandagiri 2019, 2022) to better understand abortion care seeking and experiences, including SMA experiences. Abortion is distinct from other healthcare‐seeking behavior for a variety of reasons, including abortion stigma and legality, which renders typical healthcare decision‐making frameworks less suitable for understanding the nuances of abortion‐seeking and decision‐making (Coast et al. 2018). One of the strengths of the Abortion Trajectories Framework is that it explicitly acknowledges that decision‐making about whether and how to have an abortion is not necessarily linear and is shaped by a multitude of barriers and facilitators to abortion care that individuals encounter starting from pregnancy detection. This framework is particularly useful for understanding SMA, where access to and models for SMA care are heavily determined by the assistance potentially available through accompaniment groups, telehealth providers, community health workers, or other actors along the abortion trajectory (Berro Pizzarossa and Nandagiri 2021). Other studies that seek to understand SMA decisions and care could benefit from the use of the abortion trajectories framework at conception or analysis.

As with all research, these results should be viewed in light of the limitations of our study. Despite efforts made to reassure participants, not all SMA users may have felt comfortable fully sharing aspects of their abortion decision, and social desirability bias, particularly related to abortion and self‐care, may have influenced what participants shared. Because this analysis focused on SMA users’ perspectives, there may be a variety of structural factors that influence abortion decision‐making but were not directly articulated by participants, such as the legal status of SMA or women's autonomy in decision‐making within patriarchal family structures (Gupta 2022; Kumar and Maruthakutti 2015). Our findings are not nationally or regionally representative, and differential recruitment rates in our study sites may have resulted in our findings overrepresenting the factors that influence abortion‐seekers in rural communities. However, our study has many strengths, including the variety of recruitment strategies used, particularly the community‐based and online recruitment, which allowed for a sample that included some who never sought facility‐based care during their abortions. Due to connections built by community‐based organizations serving as recruitment sites, this study includes experiences of socially marginalized individuals in India who are more likely to experience discrimination within the health system and are often underrepresented in research. This study also included the experiences of unmarried women and had a geographic spread across several states in India. To our knowledge, this is one of the largest qualitative studies documenting SMA decision‐making in India to date.

## CONCLUSION

Participants in our study reported a wide variety of influences on their pathways to SMA—sometimes simultaneously reporting a desire for self‐managed care as well as the lack of desirability of facility‐based care. Participants consistently noted the role of stigma as a factor in deterring facility‐based care, while also lacking comprehensive abortion counseling about medication and procedural abortion options. Addressing provider's negative attitudes towards abortion and abortion patients could make facility‐based care a better option for abortion seekers. To support people having self‐managed abortions in India, expanding options for information and support as well as enhancing task‐shifting efforts in the provision of abortion care and counseling to community‐based providers, could dramatically improve the quality of information and support abortion‐seekers could access.

## CONFLICTS OF INTEREST STATEMENT

The authors have no conflicts of interest to report.

## Supporting information



Supporting Information

## Data Availability

Participants did not give permission for transcripts of their interviews to be shared in a publicly available repository. As such, subsets of anonymized data may be available upon reasonable request pending review by the lead authors and coprincipal investigators.
