# Supplementary material for: “I Was Confident From the Bottom of My Heart That I Will be Fine With These Medicines”: Qualitative Analysis of Decision‐Making Around Self‐Managed Abortion Trajectories in India
Source: Stud Fam Plann. 2025 May 14;56(4):715–37. doi: 10.1111/sifp.70015 (PMC12700130; doi:10.1111/sifp.70015)
Supplement: Supplementary file 1 — Supporting Information [file SIFP-56-715-s001.docx]

**APPENDIX** I

**IDI Guide - English**

**Understanding medication abortion self-use in India**

**In-depth interview guide: SMA user**

1. **Introduction**

Different people may have different opinions about ending a pregnancy. Let’s think about your community.

- 1. How does your community view abortion?
  2. What do you think women in your community do when they have questions about abortion or post-abortion care?
     1. Where do they go? Whom do they go to?
     2. Why is that?
     3. What methods do they use for abortion?
        1. Which method do you think is most common and why?

1. **Decision-making within the family**

You shared that you had an abortion in the last two years - ___________(*specify time period based on information in the screening tool*). I would like to ask you about your living situation and decision-making within your house and family at that time.

- 1. Who were the people you were living with (i.e., nuclear family members, extended family members, living by oneself)?
  2. What was your relationship status?

Could you tell me a little bit about how decisions were made within your house? Some people make decisions in consultation with a partner or family member, while others may make decisions on their own.

For each of these situations, can you share how decisions were made in general:

- 1. Child raising/decision for running a household
  2. Usually, who made the decisions about this?
  3. What was your role (if any) in making decisions about this?
  4. What was the role of others (partner/family members) in these decisions?
  5. Managing overall finances
  6. Usually, who made the decisions about this?
  7. What was your role (if any) in making decisions about this?
  8. What was the role of others (partner/family members) in these decisions?
  9. How do you think these roles around decision-making came to be?
  10. How do you feel about your role in these decision-making processes?

1. **Pregnancy-related**

Thank you. Now, shall we talk about your experience with the pregnancy in ________ (*refer to screening tool*).

- 1. How did you find out you were pregnant (i.e., missed period, pregnancy symptoms, at home pregnancy test, etc)?

1. When was this?
   1. How did you confirm the pregnancy?
2. How many weeks pregnant were you when you confirmed your pregnancy?
3. By how many days/weeks had you missed your period when you confirmed your pregnancy? (*note to interviewer: confirm with the respondent based on their response: So to confirm, your period was delayed by ___ weeks when you confirmed your pregnancy. Is that correct?*)
   1. Did you tell anyone about the pregnancy?
   2. [*If yes*] whom did you tell? Why did you tell this person? (*note to interviewer: ask for each person the respondent shared their pregnancy with*)
   3. [*If no*] why not?
   4. How did you feel when you recognized your pregnancy? What did you think about your pregnancy?
4. Did you want to continue with your pregnancy?
5. Why/why not?
6. (*If respondent shared pregnancy news with others*) What about the person/people you shared news about your pregnancy with – how did they feel about this pregnancy? *(ask about each person the respondent mentioned)*
7. (*If respondent did not share with anyone*) What about others in your life (partner/family members) – how do you think they may have felt about this pregnancy if they had known? *(ask about each person the respondent mentioned)*
8. Did you consult/talk to anyone else apart from family members about your pregnancy? (probe: ASHA/friend/doctor/others) . What made you choose this person(s)?
   1. What did you do next?
   2. What did you decide about continuing your pregnancy?
      1. By how many weeks had you missed your period when you made this decision? (*note to interviewer: confirm with the respondent based on their response: So to confirm, your period was delayed by ___ weeks when you made this decision. Is that correct?*)
   3. How was the decision made?
   4. What role would you say your:
9. Partner played in making this decision?
10. Your family members in making this decision?
11. Others in making this decision?
12. (If others played a role) Do you think your decision would have been different if it was only up to you? How different would it have been?
13. **Methods for ending pregnancy**

Now, let’s talk about different ways to end one’s pregnancy.

- 1. When you decided to end your pregnancy, what ways/options did you think about?
  2. How did you learn about this/these method(s)?
  3. Did you ask anyone for information about how to end a pregnancy?
     1. Whom did you ask? (Partner/family member/friend/doctor/ASHA/other)
     2. What did you learn?
     3. By how many weeks had you missed your period when you learnt about this/these method(s)? (*note to interviewer: confirm with the respondent based on their response: So to confirm, your period was delayed by ___ weeks when you learnt this. Is that correct?*)
  4. Did you feel differently about your decision to end your pregnancy based on what you learnt? What was different?
  5. How did you decide about what method(s) to use to end your pregnancy?
  6. Why did you decide to use this method(s)?
  7. Did you try any/all of these methods? (probe about each method and what happened, what the outcome was, and how many weeks pregnant they were when trying)
  8. How did you feel about trying this/these method(s)? What about after you tried?
  9. Did you continue to be pregnant after trying any of these methods? What did you do then?
  10. What did you do next? (*If respondent used combipack/miso/other pills – go to V; If respondent went to an informal provider – go to VII)*

1. **Procurement of abortion pills**

Thank you for sharing your journey with me so far. Now, let’s talk about your decision to use the combipack/misoprostol/other pills and what that experience was like. We will refer to these as abortion pills.

- 1. How did you get the abortion pills? (probe: where?)
  2. Who brought/bought the abortion pills?

*[If someone else]*

- 1. Why did this person be the one to get the pills?
     1. Did you want to go get the pills? Why or why not?
  2. What do you know/think happened when this person went to the pharmacist/seller? (what information did they give/what were they asked) What information do you think this person was given (probe: how to take pills, what physical symptoms to expect, side effects, when to seek medical care, other)
  3. How many pills were you given?
  4. Do you feel like they were given enough information about the abortion pills?
  5. Do you know how much they paid for the abortion pills?
  6. Did they tell you how they felt talking to the pharmacist/seller? How do you think they felt?
  7. Do you feel like they adequately explained the information they were given to you?
     1. Was there any information you wanted but were not given/could not ask?
        1. Did you attempt to seek this information elsewhere?

*[If respondent went]*

1. Why were you the person who went to get the pills?
2. How would you describe the place you went to get the abortion pills?
3. What did you have to tell the pharmacist/seller in order to get the abortion pills? (what information did you give/what were you asked)
4. How many pills were you given?
5. What information were you given about taking the abortion pills?
   1. How to take the pills
   2. What physical symptoms to expect
   3. Possible side effects
   4. Under what situations to seek follow-up care
   5. Where to go for follow-up care
   6. Other
6. Do you feel like you were given enough information about the abortion pills?
   1. Was there any information you wanted but were not given/could not ask?
   2. Did you attempt to seek this information elsewhere?
7. How much did you pay for the abortion pills?
8. How did you feel talking to the pharmacist/seller?
   1. What other challenges did you face when trying to access the abortion pills?
9. **During the process**

Now, let’s talk about what you did after you got the pills.

1. What were your feelings and expectations taking the abortion pills? What were you worried about?
2. What did you do to prepare for the abortion process (taking the pills)?
   1. Time off from work?
   2. Childcare?
3. Did you have anyone with you for support while taking the pills?
   1. During the process?
4. Can you describe the experience/process?
   1. How many pills did you take? (*note to interviewer - if they did not take all 5 pills:* Why did you take only ___ pills?)
   2. What symptoms did you experience?
   3. How many days did you experience symptoms?
   4. How did you manage your symptoms?
   5. How did you feel during the process?
   6. Were you concerned about symptoms or complications at any point?
      1. Why were you concerned?
      2. Did you talk to anyone about these concerns?
      3. Did you seek care for your concerns?

*[If respondent sought care]*

28. Where did you receive care?

- - 1. What did the provider tell you about your complications?
    2. How did you feel about seeking care from this provider?

*[If respondent did not seek care]*

1. Why did you decide not to seek care?
   1. What did you do next? (*Go to VIII)*
2. **Informal provider services**
3. You went to _____________ to ____________. How would you describe this facility/provider?
   1. By how many weeks had you missed your period when you went to this provider? (*note to interviewer: confirm with the respondent based on their response: So to confirm, your period was delayed by ___ weeks when you went to this provider. Is that correct?*)
   2. What did you like about this facility/provider?
   3. What did you not like about this facility/provider?
   4. What did this facility/provider do?
      1. What medications were you given?
      2. What procedure(s) were followed?
      3. How much did you pay this provider?
   5. What kind of treatment did you receive?
   6. How did you feel going through this experience?
   7. Were you concerned about symptoms or complications at any point?
      1. Why were you concerned?
      2. Did you talk to anyone about these concerns?
      3. Did you seek care for your concerns?

*[If respondent sought care]*

1. Where did you receive care?
   1. What did you do next? [If the respondent took medications, go to section V]

*[If respondent did not seek care]*

1. Why did you decide not to seek care?
   1. What did you do next? [If the respondent took medications, go to section V]
2. **After the process**
3. What information and/or support would you have liked during this process?
4. How did you manage your bleeding (ie. use pads/cloth/other)?
   1. Did you dispose this? How and where did you dispose it?
5. When/how did you know the process was complete and successful?
6. Looking back, would you have liked to have recognized your pregnancy earlier?
   1. What do you think delayed you?
   2. How do you think recognizing your pregnancy earlier would have changed things for you?
7. *(If others were involved in decision-making)* Looking back, do you think your decision would have been different if your partner/family member/others were not involved?
   1. How do you think it might have been different?
      - 1. Looking back, did you feel prepared for the symptoms and side effects you experienced during the abortion process?
        2. Would you have preferred to go to a registered medical provider/approved hospital for your abortion? Why or why not?
8. **Post-abortion contraception**
9. What did you think about using contraception after this experience?
10. Did you/your partner want to use contraception?
    - 1. Why/why not?
11. Did you seek any information about contraception services after your abortion?
    - 1. [*If yes*] Where did you obtain this information?
12. Did you get contraception?
    - - 1. *[If yes]* Was this the method you wanted?
           1. What method would you have wanted?
           2. Why didn’t you get the method you wanted?
        2. *[If no]* Why not?
13. **Improvements/recommendations**
14. What would have improved your overall abortion experience?
15. What kinds of support throughout the process would have been helpful?
16. If you had a friend who is pregnant and wants to have an abortion, what would you tell her?
    1. If your friend was later in their pregnancy than you were, say at 12 weeks, what would you suggest they do?

Is there anything else you would like to tell me about your experience?

**Closing**

Thank you so much for your time and willingness to participate. Those are all the questions I have for you today. Do you have any other questions about the interview?

Appendix II

| **T1 Recruitment sample of self-managed abortion users in India who participated in in-depth interviews between January-August 2022** | | | | | |
| --- | --- | --- | --- | --- | --- |
| Study recruitment method | Clinic-based recruitment among those seeking post-abortion care | Accredited Social Health Activists (ASHAs) | Rural community-based clinic client sampling frame | Online | Total |
| Total approached | 49 | 28 | 14 | 36* | 127 |
| Spam/invalid (online only) | N/A | N/A | N/A | 8 | 8 |
| Refused to participate | 29† | 4 | 8 | N/A | 60 |
| Screened out pre-interview due to ineligibility |  | 0 | 1 | 18 |  |
| Lost to follow-up | 6 | 0 | 0 | 7 | 13 |
| Ineligible upon review of the interview | 0 | 2 | 1 | 0 | 3 |
| **Total sample** | **14** | **22** | **4** | **3** | **43** |
| * For online participants, this applies to all who filled out a consent form † Data collectors did not record whether those approached refused or were ineligible. | | | | | |

**Appendix III**

| **T2 Reflexivity Statement** | |
| --- | --- |
| Study conceptualization | The research questions for this study emerged from a stakeholder meeting about gaps in abortion research in India. During that meeting, the mostly India-based stakeholders identified the need to further investigate the perspectives of self-managed abortion (SMA) users, which became the basis of this study’s aim and justification for funding from the Packard Foundation.  One of the co-principal investigators (PIs) of this study (SC) is from India and has professional experience with sexual and reproductive health in India. This author developed the research questions after reviewing the gaps in SMA evidence in India and feedback on feasibility and suitability from subject matter experts. During this time, subject matter experts facilitated connections between Ibis Reproductive Health (Ibis) researchers and future study recruitment sites. These initial meetings also further developed the study’s research questions. The study was then designed to reflect each partner’s approach and input on study design, such as recruitment methodology, data collection materials, and standard operating procedures. |
| Data acquisition and analysis | Ibis researchers drafted the study instruments, which benefitted from critical partner review to ensure the in-depth interview guides addressed research gaps, considered relevant context, and used appropriate terminology. As each recruitment site had highly skilled researchers, capacity building or strengthening was not requested within the study partnership. Study coordinators at each recruitment site oversaw data collection and shared the de-identified data with Ibis researchers. They also provided support around quality assurance as the translated interview transcripts were received. All partners hold access and ownership rights to the de-identified data stored on a secure server managed by Ibis. |
| Data interpretation | Due to budget and capacity constraints, and despite initial plans to hire an India-based researcher, the coding was completed by Ibis researchers. During data analysis, Ibis researchers took on the initial analyses with the full study team providing iterative feedback. This dynamic, intended to distribute labor according to each team’s availability and financial support, may have alleviated certain administrative burdens from the India-based researchers at the cost of an outsized influence from researchers outside the study contexts.  Ibis researchers presented preliminary analysis to the study partners for discussion to ensure accurate contextualization and alignment on interpretation. Following those meetings, Ibis researchers began working on dissemination materials for various audiences, prioritizing resources for the communities where research was conducted. |
| Drafting and revising for intellectual content | Following data analysis and once there was shared agreement on data interpretation, all co-authors contributed to scientific papers and materials that the teams based in India are using for community outreach and client engagement. For peer audiences, the study partners have presented at local and international convenings to share study results with relevant stakeholders and local policymakers. |
| Authorship | Two early career researchers completed the majority of the coding, code summaries, and preliminary manuscript drafting and are listed as first and second authors (CB and CM). Subsequent authorship positions acknowledge the leadership team at recruitment who provided invaluable input and guidance during the research and editing process. Contributions to the study that did not merit full authorship are noted in the acknowledgement section. |
| Training | The India-based recruitment site teams, composed of senior researchers, already possessed substantial research expertise and did not require additional training. |
| Infrastructure | One potential opportunity for these findings is to better support people who self-manage and seek clinical follow-up care, but it is too early to determine how this study may contribute to improvements in local infrastructure. |
| Governance | Participants were assured anonymity in the consent forms, meaning their names and any other identifying details would not be linked to their responses. Any contact or identifying details the recruitment teams collected were not shared with the US-based team and was immediately destroyed upon completion of data collection. To further protect confidentiality, all study documents, including interview transcripts, were stored electronically in password-protected computers on secure encrypted hosting services, accessible only by the research team. Additionally, voice recordings from interviews were transcribed and de-identified before translation and analysis, ensuring the original audio file and final transcript would not contain any personal information. |
